# The CALM trial protocol: a randomised controlled trial of a guided self-help cognitive behavioural therapy intervention to reduce dental anxiety in children

**DOI:** 10.1186/s13063-022-07046-6

**Published:** 2023-01-06

**Authors:** Zoe Marshman, Helen Rodd, Caroline Fairhurst, Jenny Porritt, Bhupinder Dawett, Peter Day, Nicola Innes, Christopher Vernazza, Tim Newton, Sarah Ronaldson, Liz Cross, Jennie Ross, Sarah R. Baker, Catherine Hewitt, David Torgerson, Hannah Ainsworth

**Affiliations:** 1 School of Clinical Dentistry, University of Sheffield, Sheffield, UK; 2 York Trials Unit, Department of Health Sciences, University of York, York, UK; 3 Centre for Behavioural Science and Applied Psychology, Sheffield Hallam University, Sheffield, UK; 4 Leeds Dental Institute, University of Leeds, Leeds, UK; 5 School of Dentistry, Cardiff University, Cardiff, UK; 6 School of Dental Sciences, Newcastle University, Newcastle upon Tyne, UK; 7 Faculty of Dentistry, Oral & Craniofacial Sciences, King’s College London, London, UK; 8 Richmond Dental Care, 427-429 Richmond Road, Sheffield, UK

**Keywords:** Child dental anxiety, Randomised clinical trial, Primary dental care, Cognitive behavioural therapy intervention, Behaviour management

## Abstract

**Background:**

Globally, around 13% of children experience dental anxiety (DA). This group of patients frequently miss dental appointments, have greater reliance on treatment under general anaesthesia (GA) and have poorer oral health-related quality of life (OHRQoL) than their non-dentally anxious peers. Recently, a low-intensity cognitive behavioural therapy (CBT)-based, self-help approach has been recommended for management of childhood anxiety disorders. A feasibility study conducted in secondary care found this guided self-help CBT resource reduced DA and a randomised controlled trial was recommended. The present study aims to establish the clinical and cost-effectiveness of a guided self-help CBT intervention to reduce DA in children attending primary dental care sites compared to usual care.

**Methods:**

This 4-year randomised controlled trial will involve 600 children (aged 9–16 years) and their parent/carers in 30 UK primary dental care sites. At least two dental professionals will participate in each site. They will be assigned, using random allocation, to receive the CBT training and deliver the intervention or to deliver usual care. Children with DA attending these sites, in need of treatment, will be randomly allocated to be treated either by the intervention (CBT) or control (usual care) dental professional. Children will complete questionnaires relating to DA, OHRQoL and HRQoL before treatment, immediately after treatment completion and 12 months post-randomisation. Attendance, need for sedation/GA and costs of the two different approaches will be compared. The primary outcome, DA, will be measured using the Modified Child Dental Anxiety Scale. Scores will be compared between groups using a linear mixed model.

**Discussion:**

Treating dentally anxious patients can be challenging and costly. Consequently, these children are frequently referred to specialist services for pharmacological interventions. Longer waiting times and greater travel distances may then compound existing healthcare inequalities. This research will investigate whether the intervention has the potential to reduce DA and improve oral health outcomes in children over their life-course, as well as upskilling primary dental healthcare professionals to better manage this patient group.

**Trial registration:**

This clinical trial has been registered with an international registry and has been allocated an International Standard Randomised Controlled Trial Number (ISRCTN27579420).

## Administrative information

Note: the numbers in curly brackets in this protocol refer to SPIRIT checklist item numbers. The order of the items has been modified to group similar items (see http://www.equator-network.org/reporting-guidelines/spirit-2013-statement-defining-standard-protocol-items-for-clinical-trials/)

**Table:** 

Title {1}	The CALM trial: the clinical and cost-effectiveness of a guided self-help cognitive behavioural therapy intervention to reduce dental anxiety in children
Trial registration {2a and 2b}.	Prospectively registered with ISRCTN Registry (Trial ID: ISRCTN27579420 on 17/11/2021: https://www.isrctn.com/ISRCTN27579420)
Protocol version {3}	Version 3.0; date: 06/07/2022
Funding {4}	This project is funded by the National Institute for Health and Care Research (NIHR) Health Technology Assessment (HTA 131805). The views expressed are those of the authors and not necessarily those of the NIHR or the Department of Health and Social Care
Author details {5a}	Zoe Marshman ^1^, Helen Rodd ^1^, Caroline Fairhurst ^2^, Jenny Porritt ^3^, Bhupinder Dawett ^1^, Peter Day^4^, Nicola Innes ^5^, Christopher Vernazza ^6^, Tim Newton ^7^, Sarah Ronaldson ^2^, Liz Cross ^1^, Jennie Ross ^8^, Sarah R Baker ^1^, Catherine Hewitt ^2^, David Torgerson ^2^, Hannah Ainsworth ^2^. ^1^ School of Clinical Dentistry, University of Sheffield, UK ^2^York Trials Unit, Department of Health Sciences, University of York, UK ^3^Centre for Behavioural Science and Applied Psychology, Sheffield Hallam University, UK ^4^Leeds Dental Institute, University of Leeds, UK and Community Dental Service, Bradford District Care NHS Foundation Trust, Bradford, UK ^5^School of Dentistry, Cardiff University, UK ^6^School of Dental Sciences, Newcastle University, UK ^7^Faculty of Dentistry, Oral & Craniofacial Sciences, King’s College London, UK ^8^ Richmond Dental Care, 427-429 Richmond Road, Sheffield, UK
Name and contact information for the trial sponsor {5b}	Sheffield Teaching Hospitals NHS Foundation Trust, Clinical Research & Innovation Office, Room D49, D Floor, Royal Hallamshire Hospital, Glossop Road, Sheffield, S10 2J, UK. Tel: +44 114 271 2550 Email: sth.researchadministration@nhs.net Website: www.sheffieldclinicalresearch.org
Role of sponsor {5c}	The sponsor and funding body were not involved in the study design; collection, analysis and interpretation of data; the writing of the report; or the decision to submit the article for publication.

## Introduction

### Background and rationale {6a}

Dental anxiety (DA) is common with a prevalence of 13% in adolescents globally [1]. Children with DA are more likely to miss or delay dental appointments until they experience pain or infection. Consequently, children with DA have worse oral health and oral health-related quality of life (OHRQoL) than their non-dentally anxious counterparts [1, 2]. Dental anxiety often continues into adulthood and anxious children are more likely to become symptomatic users of dental services when adults, if their DA is not addressed [3, 4].

Treating dentally anxious patients can be challenging and costly [5]. Basic techniques such as ‘tell-show-do’ may be insufficient to manage children’s DA with the need for some to be referred by their primary dental practitioner to specialist paediatric services for treatment with pharmacological interventions (sedation, general anaesthesia [GA]) [6, 7]. Referrals for pharmacological interventions can result in children with DA having to wait longer, prolonging symptoms and needing to travel further for dental treatment [8]. As such, this creates additional potential barriers to dental care and can contribute to further increasing healthcare inequalities [9, 10]. Children who receive dental treatment under GA also continue to be at high risk of poor oral health and DA in later life [11]. Dental extractions remain the most common reason for a hospital admission for children in England. In 2018/19, there were 59,011 admissions to hospital for dental extractions in children (0–19 years) costing around £50 million, with DA being one of the most common reasons why the dental treatment could not be performed in primary dental care [12, 13].

Greater effort should therefore be directed towards interventions that can reduce a patient’s DA in the long term [6]. Psychological interventions, such as psychologist-led cognitive behavioural therapy (CBT), have shown promising results in reducing DA in adults in terms of effectiveness, acceptability and long-term benefits [14, 15]. However, the costs and availability of expert psychologists prohibit its widespread use and it is not feasible for primary care dentists to deliver complex psychological interventions within the UK National Health Service (NHS) [16]. To improve access to CBT in primary care a ‘stepped care’ approach can be used. This involves offering children the least intensive form of CBT in the first instance and ‘stepping up’ CBT interventions/support for children whose anxiety does not respond to these low-intensity treatments [17]. The stepped care approach has been found to be effective in the management of childhood anxiety [18] and the National Institute for Health and Care Excellence (2011) recommends CBT-based self-help as part of a stepped care approach for the management of a range of anxiety disorders [19]. However, there is very limited evidence about the use of low-intensity CBT self-help interventions delivered by primary care dentists and whether they are effective at reducing children’s DA, thereby reducing the need for referral to specialist services for more complex and costly pharmacological interventions including GA [20].

Recently, a self-help CBT intervention ‘Your teeth, you are in control’ has been developed which can be delivered by dental professionals (funded by NIHR Research for Patient Benefit PB-PG-1111-26029). This intervention uses a range of evidence-based psychological techniques for the reduction of DA in children with guidance provided by dental professionals. There is a self-help guide for children aged 9–16 years with accompanying resources for parents/carers and the dental team. It was developed using a ‘person-based’ approach involving dentally anxious children, parents/carers and dental health professionals to ensure the perspectives and needs of children were taken into account. A feasibility study was conducted in which 48 children with self-reported DA were given the intervention by paediatric dentists in hospital and community care settings. Children reported a mean Modified Child Dental Anxiety Scale (MCDAS) score at baseline of 25.0 (SD 6.5) and 17.4 (SD 6.1) at follow-up, demonstrating a significant reduction in DA (mean difference 7.7, *p*<0.001, 95% CI 5.7 to 9.6, Cohen’s d ES 1.2) and an increase in health-related quality of life (using the Child Health Utility 9D instrument) (mean difference −0.03, *p*<0.05, 95% CI −0.06 to −0.00, Cohen’s d ES 0.3). Overall, there was a 66% response rate and an 86% completion rate, with high levels of acceptability for children, parents/carers and dental professionals. There was also an excellent attendance rate with 90% of dental appointments kept. This compared favourably with the 74% attendance rate of children who met the study inclusion criteria but who were not recruited. Although 80% of participants were specifically referred for a GA, only 15% of these went on to require one so the intervention may reduce the need for treatment under GA [21]. When a sample of these participants were reviewed 1 year later, 91% reported feeling less worried about dental visits than they previously did, and described changes in cognition, behaviours and feelings that allowed them to manage their anxiety better [22].

Bux et al., in a single-centre service evaluation, found it was feasible to deliver this guided CBT approach in a general dental setting and reported a significant decrease in children’s DA scores [23]. These positive findings support the need for further evaluation in a multi-centre randomised controlled trial in primary dental care.

### Objectives {7}

The main aim of this study is to establish the clinical and cost-effectiveness of a guided self-help CBT intervention to reduce DA in children attending primary dental care sites across the UK, compared to usual care.

Specific objectives of the RCT are to:

- Conduct an internal pilot trial to assess:
- Trial feasibility
- Recruitment rates (of dental sites and participants)
- Dental professional and participant engagement with the intervention
- Investigate the effect of the intervention on DA (of child and parent/carer), oral health-related quality of life (OHRQoL), health-related quality of life (HRQoL), need for referral to secondary care (for proposed sedation/GA) and dental appointment attendance, over a 12-month follow-up period
- Investigate cost-effectiveness from a dental and societal perspective
- Undertake a process evaluation

### Trial design {8}

This will be a multi-region, individually randomised, pragmatic, two-arm controlled clinical trial with an internal pilot trial. This paper will focus on describing the protocol for the outcome evaluation (the protocol for the process evaluation will be detailed in a separate publication).

## Methods: participants, interventions and outcomes

### Study setting {9}

Participants will be recruited from 30 primary dental care sites, comprising general dental practices and primary dental care community clinics. These sites will be located across the UK, mainly based in the East Midlands, South Yorkshire, West Yorkshire, North East and Wales. These five regions have been chosen as they encompass some of the most deprived areas of the UK, where the prevalence of dental caries is above average. This will ensure that a socio-economically diverse and multi-ethnic mix of participants are recruited and will allow testing of the intervention in different devolved nations to increase generalisability. Local research teams, comprising a clinical lead and research dental nurse in each of the five regions, will oversee site and participant recruitment.

### Eligibility criteria {24}

#### Inclusion criteria for primary dental care sites:

- Providing NHS dental treatment to children aged 9–16 years
- 80% of sites will have a postcode in areas in the lowest three quintiles of deprivation based on the Index of Multiple Deprivation
- No previous utilisation of the guided self-help CBT intervention with child patients
- At least two dental professionals (dentists or dental therapists) willing to be involved who are not on the specialist list for paediatric dentistry and who are providing a tier one or equivalent service
- At least one of the dental professionals must have within their scope of practice the ability to conduct the initial assessment and make a treatment plan

#### Eligibility criteria for child participants and parent/carers:

- Patients aged 9–16 years, inclusive
- Child self-reported DA
- Not requiring urgent dental treatment
- Attending for a dental assessment and found to require a course of treatment for their presenting dental condition (categorised as level one complexity by NHS England), involving at least two additional visits and within the scope of practice of all participating CALM dental professionals
- Child able to read and use written English or Welsh, required to receive the intervention and complete questionnaires
- Parent/carer able to complete consent forms (with the support of an interpreter if necessary)

#### Who will take informed consent? {26a}

Informed written agreement for sites to participate will be obtained from the principal researcher of the site. Informed written parent/carer consent and child assent will be obtained by the participating dental professionals at each site.

#### Additional consent provisions for collection and use of participant data and biological specimens {26b}

Participants will be asked to consent to the storage of all identifiable trial data for 10 years following the publication of the CALM trial final report. Anonymous trial data will be kept indefinitely. This trial does not involve collecting biological specimens for storage.

## Interventions

### Explanation for the choice of comparators {6b}

The comparator for this trial is defined as non-pharmacological ‘usual care’ and is perceived as the routine way in which children with DA are managed in primary dental settings. This usual care typically relies on a number of basic behavioural approaches. These simple techniques are described in national [24] and international guidelines [25] and include:

- ‘Tell-show-do’ where children are first told about an anxiety-inducing aspect of dental treatment, then this is demonstrated before the procedure is performed;
- ‘Reinforcement’ (usually positive) of behaviour using praise and non-verbal signals
- ‘Modelling’ where the child observes another person having dental treatment undertaken
- ‘Distraction’ which can take many forms;
- ‘Voice control’ where members of the dental team alter their tone and volume of speaking to produce desired effects, and
- ‘Enhanced control’ where a specific signal from the child allows them to communicate with the dentist

Participating dental professionals and children assigned to the comparator arm will therefore undertake a course of dental treatment as per usual care.

### Intervention description {11a}

The intervention for the trial is described as a guided self-help CBT intervention called ‘Your teeth, you are in control’ (which will be made available as paper-based and online [via a website] formats; a Welsh language version is available for Welsh-speaking participants). This is accompanied by a parental resource and will be delivered by the primary care dental professional allocated to receive the intervention training. The intervention aims to normalise the experience of DA to help reduce negative emotions (e.g. embarrassment, shame) and has the following attributes:

- Helps children, parents/carers and dental team members understand the factors that may be maintaining the child's DA
- Includes information on the dental team and basic procedures
- Describes a variety of cognitive and behavioural tools/strategies that children can use to help them feel less anxious (e.g. challenging unhelpful thinking, goal setting/graded treatment planning, relaxation exercises)
- Suggests activities to increase children’s feelings of control and self-efficacy in their ability to undertake dental treatment (including a ‘message to dentist’ and signed stop signal agreement)
- Prompts children to reflect on what went well about each visit to build a memory bank of positive experiences
- Provides users with structure and guidance on how to incorporate the use of individualised positive reinforcement techniques into dental treatment/visits
- Promotes effective communication and shared decision-making between children, parent/carer and dental team members

The intervention includes a 2-h online training package for dental professionals assigned to this arm alongside a step-by-step delivery guide. The training aims to increase clinicians’ knowledge, understanding, confidence and skills in the effective management of children’s DA through highlighting how the approach and techniques included in the intervention are theoretically informed and evidence based. Children (and parents/carers) assigned to receive the intervention will be given access to ‘Your teeth you are in control’ resources prior to starting their course of dental treatment. They will be informed on how and when to use the intervention and the treating dental professional will refer to and apply the various activities outlined in the intervention at each subsequent dental visit.

### Criteria for discontinuing or modifying allocated interventions {11b}

There are no criteria for discontinuing or modifying the CBT intervention, other than child or parent/carer choice to stop using it.

### Strategies to improve adherence to interventions {11c}

As mentioned in 11a above, dental professionals who are allocated to deliver the intervention will receive bespoke online training. Additionally, a simple paper-based guide will be provided to aid adherence to the intervention protocol. Dental professionals will complete a case report form (CRF) at each participant visit to allow monitoring of fidelity to the intervention protocol.

### Relevant concomitant care permitted or prohibited during the trial {11d}

Trial participation will not require a change to the individual’s usual healthcare uptake or use of medications. However, should a clinical need arise for secondary or specialist dental care, during the study period (e.g. need for pharmacological intervention) this will be recorded on the CRF by the primary care dental professional.

### Provisions for post-trial care {30}

All participants will be scheduled for a routine review appointment with a dental professional according to national guidelines, following completion of their initial course of treatment. Any participants who require additional specialist dental care (e.g. for orthodontic treatment or pharmacological interventions) will be referred by their dental professional according to local protocols.

There are no anticipated risks from trial participation. However, all participants/parents/carers will be provided with written details of how to make a complaint should they feel that they have suffered any harm as a result of their participation in the study.

## Outcomes {12}

The primary outcome measure is child self-reported DA. This will be measured using the Modified Child DA Scale (MCDAS) at baseline (B), end of the course of treatment (Follow-up [FU]1) and 12 months post-randomisation (FU2; this is the primary time point). The MCDAS is an eight-item measure, with each item having a score ranging from 1 (relaxed/ not worried) to 5 (very worried), which are summed to give a total score of between 8 and 40. It is one of the most widely used measures of child DA [26, 27]. Furthermore, it has been validated for use with children aged over 8 years and has demonstrated its responsiveness to detect changes in dental anxiety over time [27].

Secondary outcomes will be measured at baseline, end of the course of treatment (FU1) and 12 months post-randomisation (FU2) (unless otherwise stated) and include:

(i). Health-related quality of life (HRQoL)

Child HRQoL will be assessed using the Child Health Utility 9D (CHU9D) [28]. It consists of nine dimensions (worried, sad, pain, tired, annoyed, schoolwork/homework, sleep, daily routine and activities), each represented by a single question with five response options. The responses to each of the nine questions can be taken together as a description of the HRQoL of the child and is termed a ‘health state’. There are many different health states defined by the CHU9D descriptive system (due to different combinations of response options on each of the nine dimensions), and each unique health state has a preference weight associated with it. These preference weights give a utility value (on a 0–1 scale where 1 is perfect health and 0 is a state equivalent to being dead) and can be used in economic analysis to estimate the cost per quality-adjusted life year (QALY).

(ii). Oral health-related quality of life (OHRQoL)

Child Oral HRQoL will be assessed using CARIES-QC [29], a measure of the impact of caries validated in children aged 5–16 years. CARIES-QC contains 12 items and one global question. The items are scored on a 3-point Likert scale from 0 to 2, with a higher score indicating increased impact (possible total score range 0–24). As the measure is unidimensional, a conversion scale is available to convert the raw ordinal score to an interval score to allow accurate calculation of change scores and effect sizes.

(iii). Parent dental anxiety

Dental anxiety of the parent/carer will be assessed using the Modified Dental Anxiety Scale (MDAS) [30]. This is a five-item scale, with each item coded from 1 (not anxious) to 5 (extremely anxious) giving a summated score of between 5 and 25.

(iv). Referral and use of pharmacological approaches

The need for referral to secondary dental care services and any use of sedation or GA will be recorded on a CRF for children in both groups, along with treatment provided (e.g. prevention, restoration, extraction, local anaesthesia) throughout the 12-month follow-up period.

(v). Detail on delivery of intervention/usual care

Delivery of the intervention according to the step-by-step guide (including engagement with completion of the ‘message to dentist’) and detail on usual care provided will be recorded on a CRF by the dental professional throughout the 12-month follow-up period. These details, given by dental professionals in both groups, will be used to assess fidelity and identify any possible contamination during each participant’s course of treatment.

(vi). Attendance patterns

The number of attended and missed appointments will be recorded on a CRF by the dental professional during the course of treatment and for the 12-month follow-up period. The duration of the appointments will also be recorded. This data will be recorded on a CRF by the dental professional along with the type of dental professional seen at each visit (e.g. dentist or dental therapist).

(vii). Anchor ratings

An anchor-based approach will be used to determine the change in MCDAS that represents the minimum clinically important difference (MCID) [31]. The anchors will include the following: the single-item Global Rating of Change (GRC) outcome measure used to gain the participant’s perspective on how meaningful the change to their anxiety has been. The GRC item asks participants to rate their condition (DA) following the intervention (‘I feel much worse’; ‘I feel a little worse’; ‘nothing has changed’; ‘I feel a little better’; or ‘I feel much better’) compared with baseline [32]. Participants can then be further categorised based on whether they report that their DA has improved/not changed/deteriorated using this anchor.

The Clinical Global Impressions: Improvement (CGI-I) item will be used as an anchor to capture ‘meaningful’ change in children’s DA levels from the perspective of the dental professional [33]. The dental professional will be asked to rate the participant’s DA after the intervention compared to baseline using a 7-point response scale (1=very much improved since the initiation of intervention; 2=much improved; 3=minimally improved; 4=no change; 5=minimally worse; 6=much worse; 7=very much worse since the initiation of the intervention). These data will be recorded on a CRF by the dental professional at every visit of the course of treatment, at FU1 and FU2.

### Participant timeline {13}

The 4-year study grant commenced in September 2021. Dental practice and child recruitment commenced in May 2022. It is anticipated that recruitment will take place over 24 months. Following completion of their initial course of treatment (FU1), participants will be scheduled for a review appointment at 12 months (FU2) after initial randomisation.

### Sample size {14}

From the feasibility study [21], the MCID for the MCDAS was calculated as 5 points using the anchor-based method, and the standard deviation was 6.5. Given the small sample size of the feasibility study, this trial will be powered to detect a more conservative difference of 2.5. The clustering of patients within dental professionals’ lists within sites, using an average cluster size of 20 (patients per dental site), and an intra-cluster correlation coefficient (ICC) of 0.03 is used in the sample size calculation. To detect a 2.5 point difference, assuming 90% power, 5% alpha, SD of 6.5, average cluster size of 20, ICC of 0.03 and 20% attrition, the study would need to recruit 600 children (involving 30 dental sites and 60 dental professionals). Based on the literature, it is estimated that 80% of parents/carers who are approached for the study will agree to take part; hence, it is envisaged that 750 children will need to be approached to take part [34, 35].

### Recruitment {36}

Support for enrolment will be provided by dental professionals, as appropriate, in the form of an interpreter and multimedia participant information resources hosted on a patient-facing trial website. Our patient and public involvement and engagement (PPIE) work has suggested several ways to improve involvement of children and parent/carers including using a multimedia participant information resource, offering branded stationery appealing to children and ‘thank you’ shopping vouchers.

It should also be noted that the main trial will only progress following satisfactory outcomes from an initial internal pilot trial. This will be based on the number of dental sites open to participant recruitment, the child (and parent/carer) recruitment rate in total and by site and month, and the number and percentage of participants randomised to receive the intervention who are engaging in the intervention (based on completion of the ‘message to dentist’ component of the self-help CBT resources).

In addition to monitoring overall participant recruitment rates, we will also monitor recruitment of participants living in deprived areas, based on the Index of Multiple Deprivation quintiles derived from participants’ postcodes, to ensure those living in the most deprived areas of England and Wales are included. This will be reported along with the progression criteria and changes will be made to the recruitment strategy to improve recruitment of those living in the most deprived areas if required.

At the end of the internal pilot, the response rate for the parent/carer questionnaire, which includes the resource use questionnaire, will be determined, and the choice of approach to data collection reviewed.

### Assignment of interventions: allocation

#### Sequence generation {16a}

The randomisation sequences will be generated by a statistician in York Trials Unit, UK (YTU). Randomisation of children will be 1:1 and stratified by site using variable block sizes. The statistician will also randomly select which dental health professionals will deliver the CBT intervention or usual care.

#### Concealment mechanism {16b}

The randomisation sequence will be concealed using a secure, remote web-based randomisation service at YTU.

#### Implementation {16c}

Following the child’s initial assessment by a dental professional, site staff will enter key participant details into a dedicated Trial Management System (TMS) to confirm eligibility and then randomise the participant. The TMS will inform the site staff of the allocation, which will be communicated to the participant and an appointment made with the relevant dental professional for the subsequent treatment visits. Participating dental professionals will be informed of whether they will be delivering the intervention or usual care by a trial manager with details of appropriate training provided.

### Assignment of interventions: blinding

#### Who will be blinded {17a}

Due to the open nature of the intervention, it will not be possible to blind participants, their parents/carers or treating DPs to group allocation. Members of the research team, the statistician and health economist will not be blinded.

#### Procedure for unblinding if needed {17b}

This is an open trial so there is no procedure for unblinding.

## Data collection and management

### Plans for assessment and collection of outcomes {18a}

#### Dental data

Data from dental sites and participating DPs will be collected at baseline, and throughout the 12-month follow-up period via paper-based CRFs (see appendices). Information will be sought on: need for referral to secondary care; use of sedation or GA to complete the course of planned treatment; attendance patterns of the patients; Clinical Global Impressions Improvement score (1–7) as an indicator of the DP’s perception of the degree of improvement in the child’s DA at the end of treatment. Additionally, following recruitment, DPs will also provide baseline data on (1) the socio-demographic characteristics of participants, including age, gender, ethnicity and postcode; (2) their clinical status including: caries experience (number of decayed, missing and filled teeth (dmft/DMFT)), and previous experience of local anaesthetic, sedation or GA, and (3) information as to whether the dental professionals involved in the trial from that site had ever previously seen the patient.

#### Child participants

As described previously, children will complete three validated measures to record self-reported anxiety (MCDAS); OHRQoL (CARIES-QC) and HRQoL (CHU9D) at baseline, FU1 and FU2 [27–29].

#### Parent/carer participants

Self-administered paper questionnaires will be used to collect personal data (age, gender, ethnicity, postcode of the participating child) and outcome measures from the parent/carer of participating children at baseline, FU1 and FU2. Additionally, parents/carers will report on their own degree of DA using the validated MDAS [30]. Further costs (e.g. travel time/costs for attending appointments, time missed from school, time off work for parent/carer/carers, out of pocket expenditure on medications) will also be collected via parent/carer questionnaires (for the secondary analysis, societal perspective).

### Plans to promote participant retention and complete follow-up {18b}

Child and parent/carer participants will be offered branded stationery at follow-up timepoints and given a shopping voucher (value 10 GBP) at the final follow-up appointment to thank them for taking part.

### Data management {19}

Sheffield Teaching Hospital (STH) and YTU will hold joint data controller responsibilities and will comply with all aspects of the General Data Protection Regulation 2016 applicable in the UK from May 2018.

A bespoke trial management system (TMS) will be created for the trial which will house information on questionnaire due and return dates, as well as site and participant-level data. A unique trial identification number (Trial ID) will be generated for each participant and their parent/carer at the time of entering their details into the TMS.

Paper-based forms collecting outcome data (identified only by Trial ID and returned to YTU) will be logged and then scanned using optical scanning techniques (Cardiff Teleform). Scanned data will be 100% validated and cross-checked with the information in the management database. Once received by YTU, paper consent forms and paper data (identified by Trial ID only) will be held separately and securely in a controlled access area in locked cabinets, at the University of York. Sites will keep a paper copy of consent forms for their records. This is the only participant documentation that will be retained at the sites.

YTU will be responsible for secure data archiving, and secure data deletion.

### Confidentiality {27}

Confidentiality of participants will be protected firstly through the allocation of a unique trial ID number. There will be restricted access to any personal data as both the TMS and the data management systems are held on secure University of York servers, with access limited to specified members of YTU staff as detailed in a delegation log. Personal data will be processed under Article 6 (1) (e) (processing necessary for the performance of a task carried out in the public interest) and Special Category data under Article 9 (2) (j) (processing necessary for scientific research purposes) of the General Data Protection Regulation 2016. After the trial personal information will be stored securely until the planned deletion date.

### Plans for collection, laboratory evaluation and storage of biological specimens for genetic or molecular analysis in this trial/future use {33}

This study does not involve the collection or storage of any human material.

## Statistical methods

### Statistical methods for primary and secondary outcomes {20a}

Analyses will be conducted in accordance with YTU standard operating procedures and will be undertaken in Stata v16 or later (to be confirmed in the final report). Statistical tests will be two-sided at the 5% significance level. All analyses will be conducted on an intention-to-treat basis, including all children in the groups to which they were randomised, irrespective of deviations based on non-compliance (unless otherwise specified).

The MCDAS scores at FU1 and FU2 will be compared between groups using a covariance pattern linear mixed model, adjusting for baseline scores, other pertinent baseline covariates, time and an interaction between treatment group and time as fixed effects. Child, treating dental professional and region will be included as random effects. The adjusted mean difference in MCDAS score at each time point will be extracted with its 95% confidence interval and *p*-value; the treatment effect at FU2 will be the primary endpoint, while the difference at the end of the course of treatment (FU1) is a secondary endpoint.

As part of the CALM trial, an anchor-based approach will be used to determine the change in MCDAS that represents the minimum clinically important difference (MCID). The use of multiple, independent anchors is recommended to produce a range of MCID scores for the MCDAS based on the different anchors. The ‘anchors’ will include the GRC item, the CGI-I item and clinical outcomes, including referral to specialist services and the use of sedation or GA.

CARIES-QC and parent/carer DA will be analysed in an analogous way to that described for the MCDAS.

The need for referral to secondary care and use of sedation or GA will be analysed by mixed effect logistic regression, adjusting for baseline MCDAS score and other pertinent baseline covariates as fixed effects, and dental team member and dental site as random effects. Treatment provided (e.g. prevention, restoration, extraction), attendance patterns (attended or missed appointments) and duration of attended appointments will be summarised for the two groups.

Baseline characteristics or changes in process variables that could affect the primary outcome between participants who received the intervention and participants who received usual care will be examined. Baseline and follow-up questionnaires will be analysed using linear regression modelling to measure mechanisms of impact. Data from CRFs will be compared within and between intervention and usual care to measure aspects of implementation.

The economic evaluation will assess the cost-effectiveness of the intervention versus usual care, by means of (1) a within-trial economic analysis over the trial’s 12-month time horizon, and (2) a decision analytic model, to extrapolate beyond the trial. An NHS and personal social services perspective will be taken in the base case, with a secondary analysis exploring a broader perspective. NICE recommendations will be followed wherever possible [36]. The primary economic analysis will take the form of a cost-utility analysis, to estimate the mean differences in costs and QALYs, using the CHU9D to generate utilities. In addition, a cost-effectiveness analysis will incorporate the MCDAS score (i.e. the trial’s primary outcome) as the effectiveness measure. Analyses will be undertaken on an intention-to-treat basis, in Stata v17 or later, utilising data recorded by dental practices and via self-completed questionnaires (by participants and parents/carers) regarding participants’ outcomes, resource use and costs.

Specifically, questionnaires will be completed at baseline, at the end of the course of treatment and at 12 months. Unit costs, sourced from established databases [37, 38], will be applied to each resource item to estimate a total cost per participant. Cost estimates of the intervention will incorporate the time spent delivering the intervention and undertaking the associated training, with the costs of sedation/GA referrals also included. Further costs incurred by parents/carers of participants will be collected for the secondary analysis (e.g. time off work, travel costs for appointments). Time missed from school in relation to appointments/DA will be listed as a ‘consequence’ and compared between groups, but not formally valued in monetary terms.

QALYs will be estimated for each participant using the area under the curve approach [39]. Costs will be presented in UK GBP for the appropriate year. Mean within-trial estimates of costs and health benefits will be calculated using regression methods, and multiple imputation methods will be used to deal with missing data [40], with sensitivity analysis exploring a complete case analysis. The results will be presented in terms of incremental cost-effectiveness ratios and net health benefit at 12 months. Uncertainty will be described using confidence intervals and cost-effectiveness acceptability curves [41], and sensitivity analyses will explore the impact of underlying assumptions and key parameters of the analysis in terms of the cost-effectiveness results. A pre-specified health economics analysis plan will be agreed with the trial’s independent groups.

Depending on the trial’s findings, the economic results will be modelled beyond the time horizon of the trial, making assumptions of a longer-term impact. Prior to undertaking the within-trial economic analysis, a modelling plan will be developed. The model will use data collected from the trial (outcomes, resource use, attendance patterns, referrals), supplemented by published data. Future costs and health benefits will be discounted in line with NICE recommendations [36]. Sensitivity analyses will explore uncertainty around model parameters, with the robustness of results tested using different scenarios.

### Interim analyses {21b}

A 12-month internal pilot trial (from the start of recruitment) is being undertaken to assess trial feasibility, recruitment rates (of dental sites and participants) and engagement with the intervention. The decision to progress will be taken by the trial team in conjunction with the independent groups. There are no other formal interim analyses or stopping guidelines for this trial.

### Methods for additional analyses (e.g. subgroup analyses) {20b}

A subgroup analysis will explore whether children with lower or higher baseline DA benefit more from the intervention, by including an interaction between baseline MCDAS score and treatment group in the analysis model.

### Methods in analysis to handle protocol non-adherence and any statistical methods to handle missing data {20c}

A Complier Average Causal Effect sensitivity analysis will be presented for the primary outcome to account for non-compliance with the intervention, with engagement determined by participant self-report and dentist’s assessment including evidence of completion of the ‘message to dentist’ component of the intervention [42].

### Plans to give access to the full protocol, participant-level data and statistical code {31c}

The full protocol (and any amendments) will be freely available on the NIHR website. Once the trial is complete, de-identified individual participant data and statistical codes will be available to investigators for individual participant data meta-analyses providing this has been approved by independent review committees. Data will be available from the publication date of the main trial findings, with no end date. Proposals for use of data and requests for access should be directed to the Chief Investigator.

## Oversight and monitoring

### Composition of the coordinating centre and trial steering committee {5d}

In summary, Sheffield Teaching Hospital Foundation NHS Trust will be the study Sponsor. Marshman is the Chief Investigator (CI) and responsible for clinical elements of the trial. YTU is responsible for project management. Sheffield Teaching Hospital and the University of York will hold joint data controller responsibilities.

### Trial Management Group (TMG)

The TMG is the executive decision-making body and is responsible for the day-to-day running and management of the trial. It is led by the CI and consists of members of the YTU (trial manager, statistician), and other co-applicants on the proposal. The team meets on a monthly basis via a teleconference and plan to meet face-to-face at least once a year. A Senior Management Team from within the TMG will convene by teleconference fortnightly to closely monitor milestones and deliverables.

Feedback from meetings of the various PPIE groups including the youth forum, parent panel and PPIE co-applicants will be relayed to the TMG meetings by ZM and JP.

### Trial Steering Committee (TSC)

A TSC has been set up including an independent chair, three other independent members, as well as two PPIE representatives and representatives of the funder and the sponsor. The TSC is likely to meet every 6 months, but the committee will decide on the frequency of meetings. The committee will provide overall supervision of the trial and ensure that the study is conducted according to the protocol and within the overarching ethical framework through its independent chair.

### Composition of the data monitoring committee, its role and reporting structure {21a}

#### Independent Data Monitoring Ethics Committee (DMEC)

An independent DMEC has been formed, which will be the only group who sees the confidential, accumulating data for the trial. Reports to the DMEC will be produced by the YTU statisticians and trial manager. The DMEC will meet within 6 months of the trial opening; the frequency of meetings will be decided at the first meeting. The DMEC will consider data using the statistical analyses and will advise the TSC. The DMEC can recommend premature closure or reporting of the trial.

### Adverse event reporting and harms {22}

The project does involve an intervention, but it is considered ‘low risk’ with no anticipated adverse events. Based on findings from the literature on guided self-help CBT, the feasibility study and discussions with the sponsor and PPIE representatives, it has been agreed that any adverse events from the study intervention and procedures are extremely unlikely so it can be justified not to include adverse event reporting. However, protocols are in place to minimise, report and manage any clinical or governance incidents arising during the study. The trial will adhere to the Research Governance Framework and Good Clinical Practice Guidance and all research will be carried out in accordance with the current Government COVID guidelines.

Furthermore, participants are provided with written information on how to make a complaint if they feel that they have suffered any harm as a result of the study, and this will be investigated through appropriate routes.

It should also be noted that dental teams have a statutory duty of care to all patients which includes ensuring that safeguarding arrangements are in place. In any instance where there is a safeguarding concern regarding a participant or their parent, this will take precedence and anonymity will be broken to allow for appropriate reporting and/or recording of concerns.

### Frequency and plans for auditing trial conduct {23}

Trial conduct will be closely monitored by independent subgroups (as previously described) including the TMG, TSC and DMEC.

### Plans for communicating important protocol amendments to relevant parties (e.g. trial participants, ethical committees) {25}

Any proposed substantial amendments to the trial protocol will first be discussed within the Trial Management Group and, where necessary, with the Trial Steering Committee. Necessary approvals will then be sought from the Sponsor, Funder and the Research Ethics Committee prior to implementing any changes. The trial team will take responsibility for communicating protocol amendments to all trial team members and participating sites, as well as updating trial registries and any other bodies as required. Where it is necessary to inform trial participants of a protocol change, this would likely be communicated via new participant information, approved by the REC.

### Dissemination plans {31a}

#### To participants, patients and the public

A trial website has been developed, media releases will be issued and a social media presence will be maintained throughout the trial to describe the study progress. Lay summaries of the findings will be prepared to share with all those involved with the trial.

#### To dental professional organisations/dental professionals

Dental professionals will be engaged throughout the trial via dental media releases, social media accounts describing the study progress and through clinical conference presentations. A summary of the findings will be shared with all those dental professionals involved with the trial. The trial team will make the intervention resources and online training freely available. At the end of the trial, a dissemination event is planned to which policy makers will be invited and a summary briefing will be produced. Discussions will be held with developers of relevant guidance and commissioners of dental services.

The findings of the trial will be published in a peer-reviewed and open access publication and presented at national and international oral health conferences. The implications of the trial findings will also be shared with academic teaching units to ensure the impact of undergraduate and postgraduate teaching is maximised.

## Discussion

This study brings together a team with expertise in the areas of child oral health, psychology, trials methods, medical statistics, health economics, primary dental care, process evaluation and PPIE. However, from the outset, the research team has worked closely with children, parent/carers and PPIE members to develop the protocol, and this aspect warrants further discussion. The PPIE work with children, which been ongoing since the outset of this programme of work, has included group discussions and one-to-one meetings with 20 children with different experiences of DA attending primary dental care settings. Children described how worried they felt about going to the dentist, the sights, smells, sounds and thoughts about what treatment they would need and how painful it might be. They described wanting to let the dental professional know how anxious they felt but also feeling embarrassed to admit this and feeling there was nothing the dentist could do to help them. They welcomed the idea of a resource given to them by the dentist, the opportunity to complete a ‘message to dentist’ proforma and being given more choice and control about what happened during the appointment. Some children said they preferred not to have a lot of reading to do so would like both paper-based and multimedia study information available. To thank participants for their time taking part in the trial, children would welcome a thank you voucher and branded small gifts.

Discussions were held with ten parent/carers of children with DA. Parent/carers also welcomed this study and the opportunity to test an intervention of this kind. They described frustration at not feeling able to support their own child before, during and after visits. Most parent/carers described how they themselves were dentally anxious; some parent/carers described trying to hide their DA from their child but others described talking openly about it in front of their child. Parent/carers felt that the inclusion of a measure of their own DA was useful and this may influence how well the child intervention worked. PPIE representatives endorsed the parent/carer’s view that including a measure of adult dental anxiety would be beneficial. As a consequence, the design of the study was changed to include recruiting parent/carers to complete measures of their DA. Furthermore, PPIE representatives suggested a multimedia information resource may help parent/carers with limited ability to read English to understand the trial so multimedia information will be provided. The views of children, parent/carers and PPIE representatives were obtained to choose the trial acronym.

Children, parent/carers and PPIE co-applicants will be actively involved throughout the trial. The trial team includes a young person co-applicant who has experience of DA anxiety and has previously been a member of a youth PPIE panel advising on other dental projects. An experienced PPIE representative will share the workload with the young person co-applicant.

In addition, a youth forum involving four children and young people has been convened. A parent panel of four parent/carers has also been set up to include parent/carers of children with DA. The forum and panel will meet on average three times per year. To date, the forum and panel have chosen the design of the trial logo, had input into the format of questionnaires and participant information resources (including the multimedia resources) and thank you gifts. As the trial progresses, the group will advise on participant recruitment (of participants with a range of socio-demographic characteristics), ways to promote questionnaire completion and drafting of lay summaries. The youth forum and parent panel will also be involved in the design of the dissemination strategy.

PPIE activities are co-ordinated by ZM and JP as the joint PPIE leads for the trial. They will provide ongoing tailored training and support throughout the trial for the PPIE co-applicants, youth forum and parent panel. ZM and JP will feed the findings of the PPIE activities into the Trial Management Group meetings and report them to the Trial Steering Committee. Guidance from the University of the West of England on evaluating PPIE in research will be followed and the Public Involvement Impact Assessment Framework used [43]. The GRIPP2 reporting checklist will be used to improve the reporting of the PPIE.

In summary, this trial has been driven by an acknowledged area of need, to reduce DA in children using a simple CBT-based intervention. Importantly, the substantial engagement of patients themselves will help to ensure the success and relevance of the planned research.

Undertaking research in primary dental care is not without its challenges, with financial concerns, staff workload and loss of clinical control being cited as reasons to prevent general dental practitioners (GDPs) taking part in research [44]. Previous large-scale trials have encountered issues with patient recruitment with participating practices taking longer than anticipated to recruit eligible patients [45]. Training in research has been highlighted as an area needed to help recruitment, thus a trial-specific training package was developed in conjunction with GDPs giving an overview of research methodology, good clinical practice (GCP) guidelines and trial-specific information. This can be accessed easily by all members of the dental team without having to undertake full GCP training through the National Institute for Health Research (NIHR) learn website which is not easily accessible to primary dental care teams who do not all have an allocated NHS email address. Having continued support from the trial team was noted by Keightley et al. [45] to help improve patient recruitment therefore practices are given a large amount of backing through practice visits, congratulatory emails upon patient recruitment and the trial team being easily contactable. Joint working between the trial team and GDPs included reviewing relevant trial paperwork by GDPs to check it was not too onerous and that it correlated with practice protocols [44]. Additional resources and support are even more paramount to support participating clinicians as they face a post-pandemic recovery period and an uncertain climate of contract reform in general practice.

There may be a perceived deficiency in the adoption of standardised procedures in dental practice, risking variation in intervention delivery, and also possible contamination of arms [46]. This is, however, the real picture in dental practice and understanding this can help implementation. The trial has ensured that dentists in the ‘usual care’ arm were not allowed access to CBT resources and the CBT trained staff at the practice were told not to discuss the intervention with their colleagues. The trial team also collected feedback from early adopting practices. This feedback can aid the trial team in providing guidance to new oncoming sites [46].

## Trial status

The protocol described in this paper is dated 06/07/2022 (version 3). The first participant was recruited to the trial on 16/05/2022 and recruitment is anticipated to be completed in February, 2024.

## Abbreviations

CARIES-QC: A measure of child oral health-related quality of life
CBT: Cognitive behavioural therapy
CHU9D: Child Health Utility-9D—a measure of child health-related quality of life
CI: Chief Investigator
CGI-I: Clinical Global Impressions: Improvement
CONSORT: Consolidated Standards of Reporting Trials
CRF: Case report form
DA: Dental anxiety
DMFT: Decayed, missing and filled permanent teeth
DP: Dental professional
GA: General anaesthesia
GCP: Good Clinical Practice
GDP: General dental practitioner
GRC: Global Rating of Change
HRQoL: Health-related quality of life
HRA: Health Research Authority
MCDAS: Modified Child Dental Anxiety Scale
MCID: Minimal Clinically Important Difference
MDAS: Modified Dental Anxiety Scale
MRC: Medical Research Council
NIHR: National Institute for Health and Care Research
NHS: National Health Service
OHRQoL: Oral health-related quality of life
PPIE: Patient and public involvement and engagement
QALY: Quality-adjusted life year
RCT: Randomised controlled trial
SDCEP: Scottish Dental Clinical Effectiveness Programme
TMS: Trial Management System
YTU: York Trials Unit
